# Supplementary material for: The causal relationship between the human gut microbiota and pyogenic arthritis: a Mendelian randomization study
Source: Front Cell Infect Microbiol. 2024 Nov 26;14:1452480. doi: 10.3389/fcimb.2024.1452480 (PMC11629706; doi:10.3389/fcimb.2024.1452480)
Supplement: Supplementary file 1 [file DataSheet1.zip › mendelian test/ebi-a-GCST90027478.csv_leave_one_out.pdf]

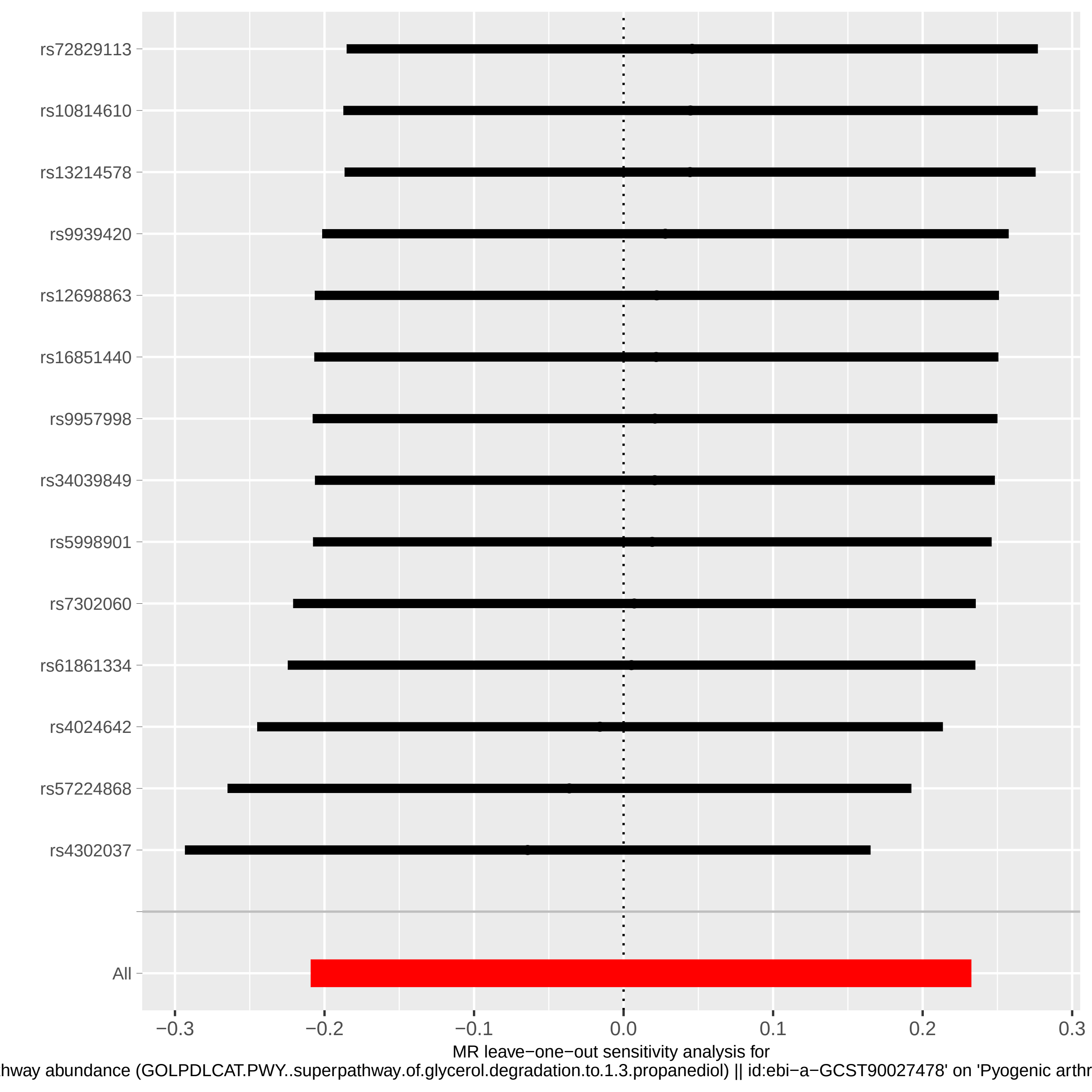
rs72829113
rs10814610
rs13214578
rs9939420
rs12698863
rs16851440
rs9957998
rs34039849
rs5998901
rs7302060
rs61861334
rs4024642
rs57224868
rs4302037
All
−0.3
−0.2
−0.1
0.0
0.1
0.2
0.3
MR leave−one−out sensitivity analysis for
hway abundance (GOLPDLCAT.PWY..superpathway.of.glycerol.degradation.to.1.3.propanediol) || id:ebi−a−GCST90027478' on 'Pyogenic arthr
